# Virome of Terrestrial Mammals and Bats from Southern Brazil: Circulation of New Putative Members of the *Togaviridae* Family and Other Findings

**DOI:** 10.3390/pathogens14040310

**Published:** 2025-03-24

**Authors:** Julyana Sthéfanie Simões Matos, Meriane Demoliner, Juliana Schons Gularte, Micheli Filippi, Vyctoria Malayhka de Abreu Góes Pereira, Mariana Soares da Silva, Matheus Nunes Weber, Marcelo Pereira de Barros, Fernando Rosado Spilki

**Affiliations:** 1 Environmental Quality Graduate Program, Molecular Microbiology Laboratory, Feevale University, Novo Hamburgo 93525-075, Brazil; vyctoriapereira@feevale.br; 2 Virology Master Program, Molecular Microbiology Laboratory, Feevale University, Novo Hamburgo 93525-075, Brazil; meriane@feevale.br (M.D.); 0206240@feevale.br (M.F.); 3 Immunology and Molecular Biology Laboratory, Faculty of Veterinary Medicine, Federal University of Rio Grande do Sul, Porto Alegre 91540-000, Brazil; julianaschons@feevale.br (J.S.G.); matheus.weber@ufrgs.br (M.N.W.); 4 Virology Master Program, Faculty of Veterinary Medicine, Feevale University, Novo Hamburgo 93525-075, Brazil; marianasilva2@feevale.br; 5 Environmental Quality Graduate Program, Zoology Laboratory, Faculty of Veterinary Medicine, Feevale University, Novo Hamburgo 93525-075, Brazil; marcelopb@feevale.br; 6 Environmental Quality Graduate Program and Virology Graduate Program, Faculty of Veterinary Medicine, Feevale University, Novo Hamburgo 93525-075, Brazil; fernandors@feevale.br

**Keywords:** chikungunya, wild animal, viral diversity, metagenomics

## Abstract

The surveillance of wildlife viromes is essential for identifying zoonotic threats within the One Health framework. This study analyzed rectal and oral swabs from 88 individuals representing 13 species as felids, wild rodents, marsupials and non-human primates in Southern Brazil using metagenomic sequencing. *Akodon montensis* (*n* = 15 individuals) and *Coendou spinosus* (*n* = 4) harbored Chikungunya virus (ChikV, Togaviridae), marking its first detection in these hosts. *Molossus molossus* (*n* = 17) presented Coronaviridae and Orthoherpesviridae, while *Eptesicus furinalis* (*n* = 1) also carried Coronaviridae. A broad virome diversity, including Togaviridae and Adenoviridae members, was identified in *Didelphis albiventris* (*n* = 43), with significant relevance to human health. Additional species, such as *Callithrix jacchus* (*n* = 1), *Leopardus guttulus* (*n* = 1), *Myocastor coypus* (*n* = 1), *Monodelphis iheringi* (*n* = 1), *Thaptomys nigrita* (*n* = 1), *Sooretamys angouya* (*n* = 1), *Brucepattersonius iheringi* (*n* = 1), and *Lasiurus blossevillii* (*n* = 1), contributed to insights into viral reservoirs. These results underscore the importance of virome studies in regions harboring high biodiversity, emphasizing genomic surveillance as a vital tool for monitoring zoonotic viruses and safeguarding global health.

## 1. Introduction

Viral metagenomics has revolutionized virology by providing culture-independent methods for detecting and characterizing viruses, especially those that cannot be cultured or isolated. This approach focuses on viral nucleic acids, offering new insights into viral ecology, including the diversity, abundance, and functional roles of viruses in different environments [1,2,3]. It is now an essential tool in genomic surveillance, enabling the rapid identification and tracking of viral mutations, which is vital for pandemic monitoring and prevention. This facilitates effective public health responses, particularly in the timely detection of emerging pathogens [3].

Genomic surveillance plays a crucial role in preventing the global spread of potential pathogens, enhancing pandemic preparedness. It has been key in identifying and characterizing viruses such as *SARS-CoV-2*, Dengue, Zika, and Chikungunya, particularly in tropical and subtropical regions [4]. Additionally, genomic analysis is critical for vaccine and antiviral therapy development. By identifying conserved viral targets, it helps design treatments that can withstand viral mutations. Mendes and Silva (2022) [5] emphasize the importance of genomic data for adaptive vaccine strategies that address new viral strains, which may evade existing immunity [5].

Monitoring arboviruses transmitted by vectors is particularly vital due to climate change and human activities that expand the range of mosquito vectors. Real-time surveillance tracks the evolution and spread of these viruses, enabling targeted interventions during outbreaks [6]. The gastrointestinal virome of wild animals also acts as a reservoir for emerging viruses, making wildlife surveillance essential to prevent viral threats to both human and animal populations [7,8]. Recent metagenomic studies have highlighted the vast viral diversity in wildlife, further underscoring the significance of this approach [4,9]. In Brazil, Pinheiro et al. [10] analyzed 58 individuals from the species *Molossus molossus* and *Molossus rufus* using metagenomic techniques and identified the presence of viral families, including Poxviridae, Mimiviridae, Retroviridae, Siphoviridae, Myoviridae, Retroviridae, Herpesviridae, Picornaviridae, Phycodnaviridae, Inoviridae, and Dicistroviridae. Duarte et al. [7] collected fecal samples from bird species (*Psittacara leucophthalmus*, *Amazona aestiva*, and *Sicalis flaveola*) and mammal species (*Didelphis albiventris*, *Sapajus libidinosus*, and *Galictis cuja*) within the Cerrado biome in Brazil, employing metagenomic techniques. Among the detected viral families, notable findings included Adenoviridae, Anelloviridae, Circoviridae, Caliciviridae, and Parvoviridae. This study also reported the presence of a novel virus belonging to the Smacoviridae family.

The aim of this study was to identify the viral families present in rectal and oral swabs of wild mammals in Southern Brazil using the metagenomic technique. This type of study in wild animals in this region is unprecedented, and such identification led to the exceptional finding of Chikungunya virus in these animals, which, until now, has not been reported in animals in the region.

## 2. Materials and Methods

### 2.1. Research Area

The study was conducted in the Sinos River Valley region, located in Rio Grande do Sul State, Brazil. The area encompasses 30 municipalities, totaling 3694 km^2^ and an estimated population of 1,440,500 inhabitants. The predominant climate is subtropical, and the landscape is largely urban, characterized by intensive industrial, agricultural, and livestock activities, with only a few remaining areas of preserved vegetation, free from anthropic interference [11].

The mammalian fauna of this region encompasses species from the Atlantic Forest and Pampa biomes, fulfilling critical ecological functions. Carnivores such as the pampas fox (*Lycalopex gymnocercus*) and the crab-eating raccoon (*Procyon cancrivorus*); rodents including the capybara (*Hydrochoerus hydrochaeris*) and the Brazilian guinea pig (*Cavia aperea*); as well as marsupials like the white-eared opossum (*Didelphis albiventris*) contribute significantly to ecosystem stability. Additionally, bats from the genera *Artibeus* and *Molossus* play essential roles in pollination and insect population regulation. However, the region also harbors wild felids at risk of extinction, including Geoffroy’s cat (*Leopardus geoffroyi*), the ocelot (*Leopardus pardalis*), the margay (*Leopardus wiedii*), the southern tiger cat (*Leopardus guttulus*), the pampas cat (*Leopardus colocolo*), and the cougar (*Puma concolor*), all categorized as vulnerable or endangered. The sampling areas were chosen due to their proximity to human occupation, intense environmental impact, and documented biodiversity. Urbanization and habitat fragmentation constitute major threats to local wildlife, highlighting the urgent need for targeted conservation strategies to ensure the persistence of these species within their native ecosystems.

**Figure 1:**
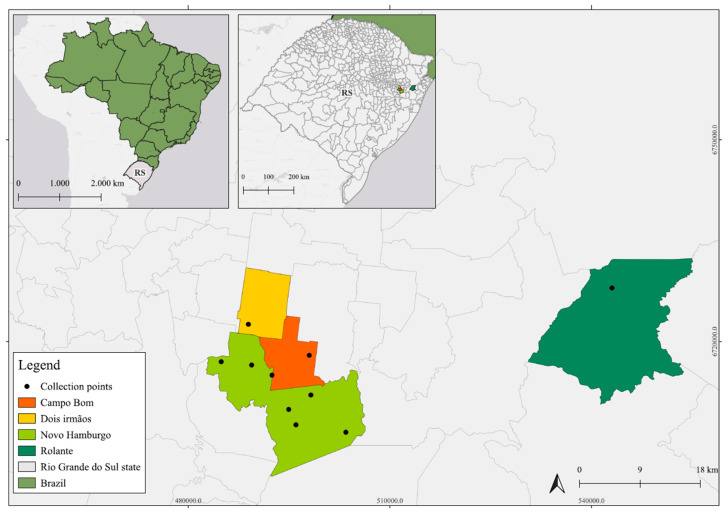
The collection locations of the animals included in this study can be seen in the municipalities of Campo Bom (Orange), Dois Irmãos (Yellow), Novo Hamburgo (Lime Green) and Rolante (Green), as indicated on the map of the state of Rio Grande do Sul (RS).

The collections were conducted in the municipalities of Novo Hamburgo, Campo Bom, Dois Irmãos, and Rolante. Complete information about the samples can be observed in Figure 1.

### 2.2. Animal Sampling

In this study, rectal and/or oral swabs were collected from the sampled animals. Swabs were collected, identified, and individually stored in tubes containing 3 mL of 0.9% sterile saline solution, then transported in an isothermal box maintained at 5 °C to the Molecular Microbiology Laboratory of Feevale University. At the laboratory, the samples were prepared in 2 mL aliquots for final storage at −80 °C. Table 1 shows the municipalities and locations where the animals were collected, as well as the number of individuals collected for each species.

For the capture of small rodents, opossums, and porcupines, two trapping sessions were conducted per month, each lasting three days. During each session, 30 Tomahawk traps (30 cm × 20 cm × 20 cm) were used, distributed along a transect of approximately 300 m, with the traps spaced approximately 10 m apart. The traps remained in place for the three days of each session. In total, the capture effort totaled 21,600 trap-days over the entire sampling period. The traps were baited with a mixture of peanuts, bananas, sardines, and sardine oil, with approximately 10 grams of bait per trap. The baits were checked and repositioned every 24 h, as needed.

Bats were captured using mist nets in the rural area of Novo Hamburgo (Table 1). Two capture campaigns were conducted at each location, each lasting approximately seven hours, totaling 14 h of capture effort.

Samples from nutria, wild felids, larger opossums, larger porcupines, and White-tufted-ear Marmoset were obtained from Environmental Rescue Centers and Agricultural Services in local municipalities. After capture, all animals were returned to their natural habitats, ensuring health parameters and welfare standards were met prior to release.

The identification of small rodents, opossums, nutria, porcupines, wild felines and White-tufted-ear Marmoset was performed based on regional descriptions, morphological analyses and consultations with the Brazilian Biodiversity Information System (https://www.sibbr.gov.br/, accessed on 31 March 2023). The identification of bats was performed through morphological analysis and support from the Bat Management and Control Guide of the State Health Surveillance Center of Rio Grande do Sul, Brazil.

In this study, 88 animals were subjected to rectal and oral swabbing, totaling 77 samples, collected between March 2022 and March 2023. The species identified across all samplings were *Didelphis albiventris* (opossum), *Coendou spinosus* (porcupine), *Myocastor coypus* (nutria), *Leopardus guttulus* (Atlantic Forest tiger-cat), *Callithrix jacchus* (White-tufted-ear Marmoset), *Monodelphis iheringi* (Ihering’s Three-striped Opossum), *Akodon montensis* (small wild rodent), *Brucepattersonius iheringi* (small wild rodent), *Thaptomys nigrita* (small wild rodent), *Sooretamys angouya* (small wild rodent), *Lasiurus blossevillii* (bat), *Eptesicus furinalis* (bat), and *Molossus molossus* (bat). Table 1 shows the municipalities and locations where the animals were collected, as well as the number of individuals collected for each species.

For the metagenomics assay, the samples were pooled based on animal species, location, sex, type of swab sample, and collection date. A total of 77 pools, each containing 1 mL and composed of samples from two to four animals, were analyzed. In cases where an animal had only one sample, it was processed individually with a 1 mL aliquot.

### 2.3. Viral Nucleic Acids Isolation, Enrichment and Sequencing

For the metagenomics assay, pools were homogenized by vortexing and filtered with a 0.45 µm membrane. Nucleic acid extraction was performed using the MagMAX™ CORE nucleic acid purification kit (Applied Biosystems, Waltham, MA, USA) with the automated KingFisher™ Duo Prime system (Thermo Fisher Scientific Inc., Waltham, MA, USA). First-strand cDNA synthesis was performed with Superscript IV (Thermo Fisher Scientific Inc.,Waltham, MA, USA), followed by second-strand synthesis with NEB Q5 polymerase (New England Biolabs, Ipswich, MA, USA), according to the manufacturer’s instructions and the protocol described by Demoliner et al. (2023) [12].

Subsequently, a shotgun metagenomic library was constructed using the Illumina DNA Prep kit (Illumina Inc., San Diego, CA, USA), following the manufacturer’s recommendations. Sequencing was conducted on the Illumina MiSeq platform using the 600-cycle V3 reagent kit (Illumina Inc., San Diego, CA, USA).

### 2.4. Viral Metagenomic Data Assembly and Sequence Analyses

Bioinformatics analyses were conducted using the online tool Genome Detective (https://www.genomedetective.com/). The Genome Detective pipeline for viral identification in metagenomics starts with raw FASTQ file input, typically from paired-end sequencing platforms like Illumina MiSeq. The process begins with quality control, where low-quality reads and adapter sequences are removed. Next, the pipeline assembles sequencing reads into contigs using SPAdes, an assembler optimized for metagenomics. These contigs are then subjected to taxonomic classification by aligning them against curated viral reference databases. Genome Detective integrates automated tools for pathogen identification, ensuring accurate recognition of viral genomes, including rare or novel strains, and supports high-throughput data processing to expedite analyses in clinical and environmental settings [13,14].

Post-classification, the pipeline offers features for downstream analysis, such as consensus genome building, phylogenetic tree construction, and the visualization of sequencing coverage and mapping statistics. These capabilities are coupled with annotation tools that refine viral genome characterization. The platform provides robust performance in metagenomics, enabling it to identify co-infections, viral subtypes, and variants within complex samples. The integration of annotation and phylogenetic tools allows for rapid insights into evolutionary relationships and epidemiological trends, making it an invaluable resource for outbreak investigations and pathogen surveillance [15].

Subsequently, for the phylogenetic analysis, complete genome sequences of Chikungunya virus and the ORF-1 region of Torque Teno virus were selected. Initially, the genome was assembled by mapping the reads from the libraries generated for each sample against the reference genome NC 004162 for Chikungunya virus and NC 040617 for the ORF-1 region of Torque Teno virus in Geneious Prime software version 2025.0.3. Next, multiple sequence alignment was conducted using the MAFFT online tool version 7 (https://mafft.cbrc.jp/alignment/server/, accessed on 10 January 2025), employing default parameters to optimize the alignment. Phylogenomic inference was then carried out using the IQ-TREE web server (http://iqtree.cibiv.univie.ac.at/, accessed on 10 January 2025), which applied maximum likelihood methods with model selection and ultrafast bootstrap approximation to ensure statistical robustness. Finally, the phylogenetic trees were formatted and refined using MEGA version 11.0.13, facilitating clear visualization and interpretation of evolutionary relationships.

For the analysis of viral diversity, a heatmap demonstrating the relative abundance of viral families present in each animal species was created. The heatmap was generated by considering both the read counts attributed to each viral family within each animal species and the frequency of occurrence of a given viral family across the respective species.

## 3. Results

### 3.1. Overview

After performing the metagenomic assay, a total of 9.831.937 reads and 2.5 Gb of information were generated.

Also, CZ ID was used to evaluate the genus found in the metagenomic. Through the analysis of the CZ ID, a total of 54 viral genera were identified in *Didelphis albiventris*, 29 in *Molossus molossus*, 17 in *Akodon montensis*, 9 in *Leopardus guttulus*, 6 in *Monodelphis iheringi*, 4 in *Coendou spinosus*, 2 in *Callithrix jacchus*, 2 in *Eptesicus furinalis*, 2 in *Lasiurus blossevillii*, 2 in *Sooretamys angouya*, 1 in *Brucepattersonius iheringi*, 1 in *Myocastor coyous*, and 1 in *Thaptomys nigrita.* A heatmap of the findings by viral family was made, and Supplementary Materials contains a table with all the genera found by animal, as well as the heatmap by viral genus created by CZ ID.

**Figure 2:**
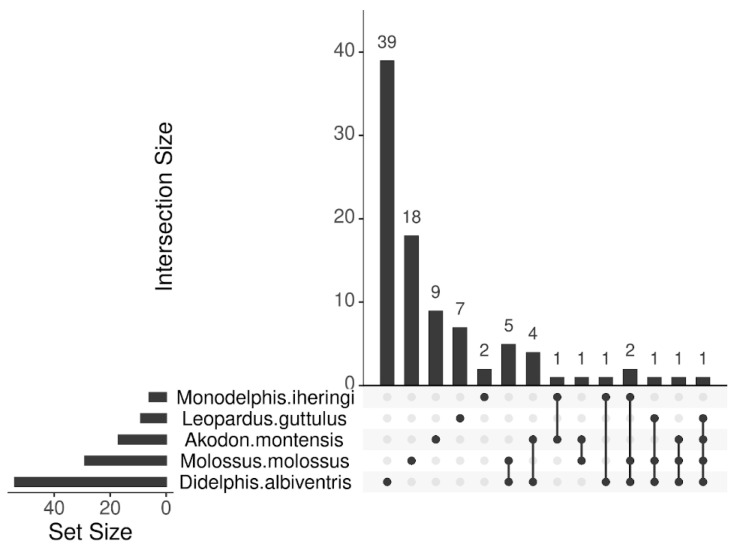
Number of viral genus identified. Horizontal bars represent the total number of genera identified by species, while vertical bars indicate the number of genera shared between the animal species. This visualization highlights the overlap and uniqueness of viral genera detected. Graphic done at RStudio version: 2024.12.1, leveraging the ‘UpSetR’ library.

Then, a graph illustrating the animal species that exhibited the highest diversity of viral genera was generated (Figure 2). Each bar in the graph represents the quantity of genera that are either unique to a specific species or shared among the analyzed species.

### 3.2. Viruses in the Studied Species

*Callithrix jacchus* (White-tufted-ear Marmoset) is an exotic species in the study area, obtained through the Environmental Rescue Center and Agricultural Services. In this study, the viral families found in *C. jacchus* were all bacteriophages belonging to the Caudovirecetes class.

The rectal swab from *T. nigrita* (small wild rodent) showed the presence of Caudoviricetes and Peduoviridae viruses. Additionally, the rectal sample from the rodent *M. coypus* (nutria) showed the presence of Togaviridae-related genomes only, and this is the first report of this viral family in this species.

Additionally, oral samples from *A. montensis* (small wild rodent) also showed the presence of Straboviridae, Autographiviridae, and Adenoviridae. The viral families common to both type of samples in this species were Togaviridae, Retroviridae, Marseilleviridae, Peduoviridae, and Caudoviricetes.

The oral swab from *S. angouya* (small wild rodent), and *B. iheringi* (small wild rodent) showed the presence of Retroviridae-related sequences, while the oral samples from *S. angouya* (small wild rodent) demonstrated the presence of Caudoviricetes, too.

In rectal samples from *M. molossus* (bat), Orthoherpesviridae, Retroviridae, Caudoviricetes, Coronaviridae, and Microviridae were detected, while oral samples only showed Caudoviricetes and Microviridae. All oral swab samples from *E. furinalis* (bat) showed the presence of Retroviridae. For this same type of sample, *L. blosevilii* (bat) showed the presence of Coronaviridae.

The rectal sample from *M. iheringi* (Ihering’s Three-striped Opossum) revealed the presence of Riboviria and Caudoviricetes. In the oral sample from the same species, Caudoviricetes and Orthomyxoviridae were detected. This is the first study of these viruses in this species.

For *C. spinosus* (porcupine), both rectal and oral samples showed the presence of Togaviridae, Retroviridae, and Caudoviricetes.

The samples from *D. albiventris* exhibited the greatest diversity in terms of the number of viral families found. Furthermore, based on Figure 2, *D. albiventris* was the species with the highest viral diversity, presenting a total of 54 viral genera. Among these, 39 genera were found exclusively in this species, while the remaining 15 genera were also detected in other animal species. The high viral diversity in *D. albiventris* is a consequence of the sample size, as this species comprises 50% of the total sampled animals, as well as its omnivorous habits.

**Figure 3:**
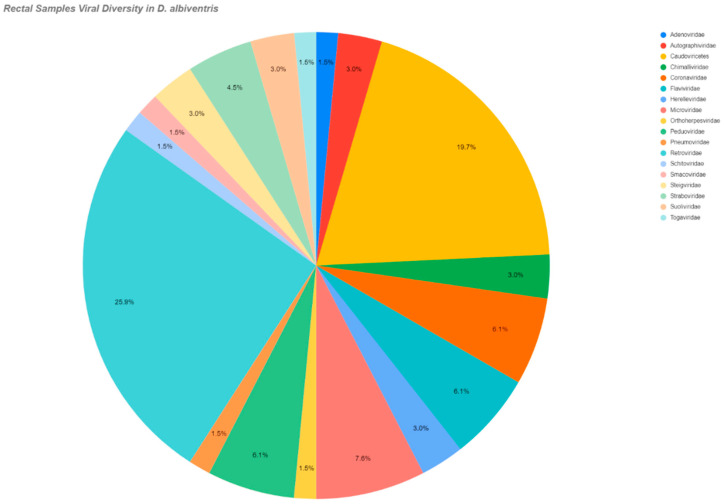
Demonstration of the viral families present in the rectal samples of *D. albiventris*, as well as the percentage of each in the evaluated pools.

**Figure 4:**
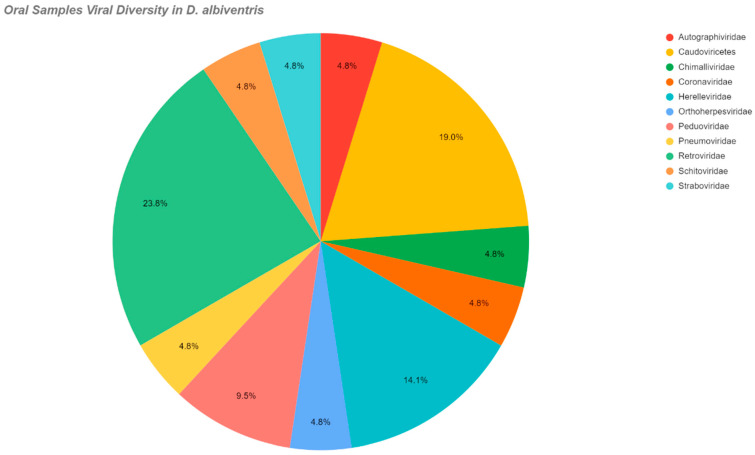
Demonstration of the viral families present in the oral samples of *D. albiventris*, as well as the percentage of each in the evaluated pools.

**Figure 5:**
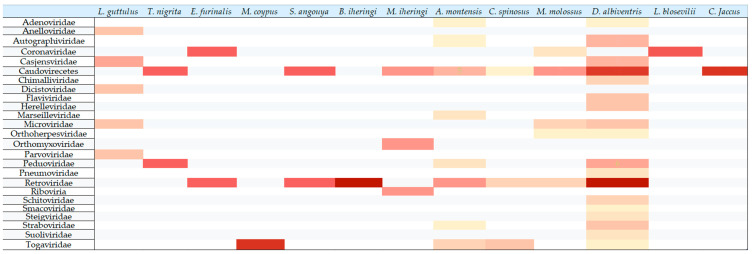
Heatmap illustrating the relative abundance and distribution of viral families identified across the analyzed animal species. The heatmap was generated using Microsoft Excel 2501. Viral families are listed on the *y*-axis, while the *x*-axis displays the corresponding animal hosts. The color intensity represents the number of viral hits detected for each family within each species, ranging from light beige (representing a minimum of 1 hit) to dark red (representing a maximum of 10,084 hits). The frequency of occurrence of each viral family among the studied species ranged from 1 to 21 occurrences. This analysis highlights the diversity and prevalence of viral families across different vertebrate hosts as determined by metagenomic sequencing.

Also, Table 2 shows the viral families or classes by species and kind of swab. Figure 3 illustrates the viral families detected in rectal swabs of *D. albiventris*, while Figure 4 presents the viral families identified in oral swabs from the same species. Subsequently, Figure 5 displays a heatmap that compares the relative abundances of viral families across the studied species. This heatmap was constructed by integrating the quantitative distribution of sequencing reads assigned to each viral family, as well as the incidence rate of detection for each viral family within the respective host species.

### 3.3. Chikungunya Virus (ChikV)

**Figure 6:**
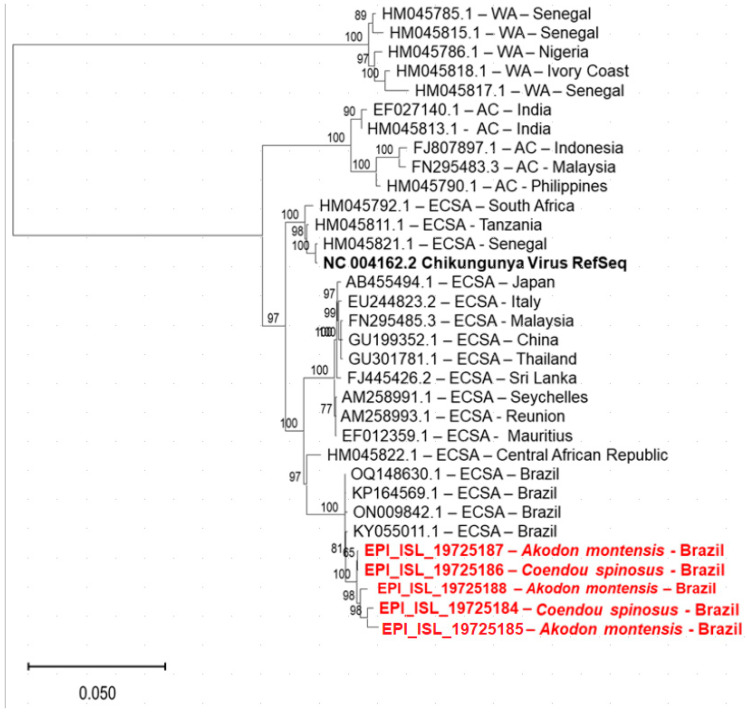
Chikungunya virus maximum likelihood phylogenetic analysis, tree model GTR+F+I, conducted using the IQ-TREE v1.6.12 web server, with 1000 bootstrap replicates. Sample sequences with whole genome and sized to 11778 to 11865 pb. Sample sequences submitted to GISAID platform. Note: AC—Asian and Caribbean Genotype; WA—West African Genotype; ECSA: East-Central-South Africa Genotype.

The Togaviridae family virus found in *A. montensis* (small wild rodent) and *C. spinosus* (porcupine) corresponds to the Chikungunya virus (ChikV), with complete genome sequences being found in pools 17, 18, 22, 23 and 24, which were selected for phylogenetic analysis. Table 3 shows the data of each sample. According to the Chikungunya Typing Tool (https://www.genomedetective.com/app/typingtool/chikungunya/, accessed on 31 March 2022), all of them were from ECSA genotype, which could be confirmed by phylogenetic analyses. Figure 6 shows the phylogenetic analyses of ChikV.

### 3.4. Anellovirus in L. guttulus

The rectal sample from *L. guttulus* revealed the presence of reads from viral families Anelloviridae, Microviridae, Dicistoviridae, Parvoviridae and Casjensviridae.

**Figure 7:**
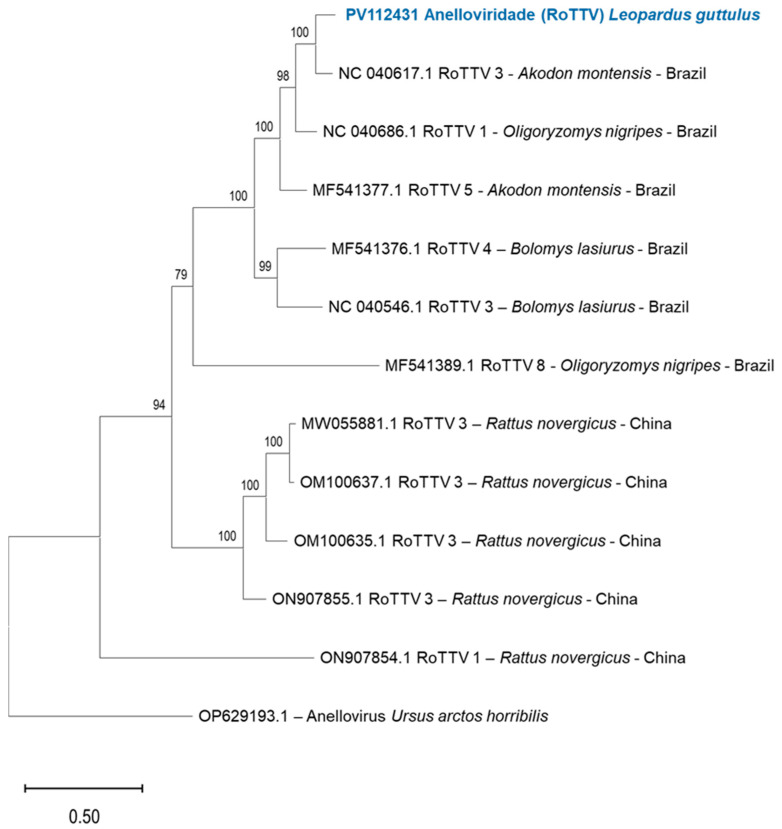
*L. guttulus* Anelloviridae virus Maximum Likelihood phylogenetic analysis performed in IQ-TREE v1.6.12 web server, applying 1000 replicates, tree model TPM2u+F+G4 and sample size with 1491pb. Sequences retrieved from NCBI Nucleotide. Sample sequence submitted to NCBI Nucleotide platform under accession number PV112431.1.

The analysis of the Anellovirus genome fragments in the *L. guttulus* sample was related to the previously identified Rodent Torque Teno Virus 3 (RoTTV-3). These fragments consisted of 5 contigs and 10 reads, with a genome coverage of 27.2% and a nucleotide identity of 86.2%. After detecting the virus through the Genome Detective online platform, blast and phylogenetic analyses were conducted to confirm its similarity to previously reported cases. Figure 7 shows the phylogenetic analysis.

## 4. Discussion

*Callithrix jacchus* (White-tufted-ear Marmoset) has become invasive in various regions of Brazil, with a high capacity for adaptation and reproduction, and there are already reports of hybrids in the country. Its adaptability to new habitats leads to competition for shelter and food with native species, potentially causing significant changes in local biodiversity. Additionally, it can be a vector of diseases that affect both other animals and humans, increasing the risk of infectious outbreaks [14]. The presence of *C. jacchus* in non-native areas can alter the ecological balance, leading to stress and competition with native species, as well as the degradation of the ecosystem in which it is introduced. The potential for disease transmission includes, in particular, zoonotic pathogens that can be transmitted to other wildlife species, domestic animals, and humans. Addressing this issue requires integrated management strategies, such as habitat preservation, control of invasive populations, and awareness campaigns to reduce human–wildlife interactions, especially in urban areas.

The White-tufted-ear Marmoset feeds on a range of items, from fruits and flowers to animal prey such as lizards and frogs, which would explain the presence of bacteriophages in both the oral and rectal swabs. Furthermore, considering the adaptability and invasive occupation of the species, as previously mentioned, this species deserves attention, given that non-human primates are already known to be hosts of many diseases [14].

All small wild rodents collected in this study belong to the family Cricetidae. According to Kane et al. [16], rodents of this family have previously been studied for RNA viruses, and among the viral families present in them is Retroviridae, which were also found in this study. Fan et al. [17] also found Retroviridae virus in small rodents. Kane et al. [16] and Fan et al. [17] also reported Adenoviridae, Marseilleviridae and Togaviridae viruses in small rodents from wild areas.

A study performed in 2023 describes Smarcoviridae and Retroviridae in samples from *D. albiventris* [18]. Some authors suggest that sequences classified as retroviruses may be proviruses that established themselves in the germline of the genus Didelphis in the past, given that this genus emerged over 4 million years ago [18,19]. While most endogenous retroviruses are defective, some may, upon activation, give rise to progenitor viruses that can infect other cells [18,20].

Bitencourt and Bezerra [21] describe that Alphavirus, a genus of the family Togaviridae, was also found in *D. albiventris*. This genus is the same found in the Didelphidae animals of this study which presented Togaviridae virus in their samples.

The family Togaviridae has previously been found in small rodents, according to Kane et al. [16]. Also, according to Matusali et al. [22] ChikV was already found in African rodents. The same authors affirm that rodents serve as virus-amplifying hosts. Petitdemange et al. [23] affirm that rodents are one of the hosts responsible for ChikV sylvatic cycles maintenance, although. Even though it’s known that rodents are ChikV hosts, this is the first report of ChikV in *A. montensis* in Brazil, as well as in *C. spinosus*.

According to the arbovirus databases for the State of Rio Grande do Sul, Southern Brazil (https://saude.rs.gov.br/arboviroses-paineis-de-dados, accessed on 20 December 2024), autochthonous cases of Chikungunya Fever in humans were reported in the study region during 2022 and 2023. Therefore, the presence of this virus in the samples is plausible. The relationship between outbreaks of human arboviruses and the circulation of these viruses in wild animals reinforces the need for continuous genomic and epidemiological surveillance. Environmental degradation and habitat fragmentation can contribute to the emergence of zoonotic diseases, as they alter vector behavior and host interactions, directly impacting public health [24].

Furthermore, as noted by Bezerra et al. [25], gastrointestinal complaints are among the clinical signs associated with ChikV pathogenesis in humans. Given the current understanding of the virus pathogenesis, it is not entirely possible to refute the potential occurrence of viremia in *Akodon montensis* and *Coendou spinosus*, nor can it be ruled out that this species may serve as a host involved in the disease cycle within the study area. However, further evaluations are necessary to verify this hypothesis. Thus, Cavalcanti et al. [26] and Diallo et al. [27] cite that the virus is maintained in a rural enzootic transmission cycle, which occurs between various sylvatic Aedes (Stegomyia) mosquitoes and animal reservoirs.

Migné et al. [28] describe that certain mammals, particularly those belonging to the orders Rodentia and Chiroptera, are considered effective reservoir hosts and/or amplifying hosts due to their significant capacity for locomotion and adaptation to new habitats. Environmental changes and rapid urbanization can facilitate contact between humans and these reservoirs, increasing the risk of zoonotic disease transmission. Conservation strategies, wildlife management, and vector control are essential to reducing these risks and mitigating the emergence of zoonoses, especially the new ones [29].

García-Romero et al. [30] further assert that the emergence and spread of infectious diseases are intrinsically linked to human–environment interactions. In this context, Dobson et al. [31] highlight that conserving biodiversity and reinforcing natural ecological barriers are essential strategies to mitigate the risk of viral spillover, thereby playing a crucial role in preventing future pandemics. Additionally, detecting these diseases in hosts not traditionally considered natural reservoirs provides valuable insights into their complex transmission dynamics, which may require targeted interventions at the levels of the environment, pathogens, and hosts. Furthermore, García-Romero et al. [30] emphasize that well-preserved habitats significantly reduce the likelihood of spillover events, while greater biodiversity is associated with lower disease prevalence among hosts, reinforcing the critical role of ecological integrity in disease prevention.

Additionally, the circulation of arboviruses typically occurs in sylvatic or urban cycles, and disruptions to the natural balance of environmental systems can increase vector abundance, create new reservoirs, or drive arboviruses to adapt in order to maintain a stable transmission cycle. The relationship between humans and various mammalian species is also recognized as a significant factor that promotes the persistence of arbovirus cycles and their hosts [32,33].

Migné et al. [28], Fraiture et al. [34], dos Santos Fonseca et al. [35] and Milich et al. [36] discuss emerging methods for sampling wild animals to detect arboviruses. These authors highlight that the use of non-invasive techniques, such as sampling saliva and feces, can facilitate the diagnosis of these viruses in animals. This approach not only reduces stress on the monitored animals and lowers costs but also expands the range of mammalian orders that can be explored for this purpose, as well.

*L. guttulus* inhabits a broad variety of habitats, ranging from dense tropical and subtropical rainforests, deciduous and semi-deciduous forests, and mixed pine forests to open savannahs and beach vegetation, both pristine and disturbed [37,38]. It can also inhabit disturbed formations, but the occurrence of this felid is limited by the presence of natural cover. Thus, it is restricted to forest or savannah mosaics and small-scale agriculture. According to Kitchener et al. [39], Rio Grande do Sul State is the type locality for this species.

Diel’s activity pattern is predominantly nocturno-crepuscular, but with a considerable amount of daytime activity. Its prey base consists mostly of small mammals, birds and lizards, with average prey size at <100 g, but does include a few larger-sized prey (>1 kg) [37,40].

Rinaldi et al. [41], evaluating the feeding habits of small neotropical felids in Brazil, found that *L. guttulus* primarily feeds on Akodontini rodents, including *Akodon montensis*. The most important food items were Akodontini rodents and Monodelphis sp. marsupials. Prey heavier than 1000 g were not detected in the diet of this felid, corroborating Seibert et al. [42] regarding prey sizes and preferences for this species. Additionally, *Oligoryzomys* sp. was also found in the diet of *L. guttulus* by [38].

Anelloviruses, such as Torque Teno Virus (TTV), have been detected in various mammals, including felines and rodents. In rodents, a thorough examination of the natural hosts of anelloviruses is crucial for understanding their host range and transmission dynamics in natural settings [43].

RoTTV was also previously found in fecal samples from *Lynx rufus* (Bobcat), as reported by Kraberger et al. [44]. These authors suggest that the presence of these viruses in fecal samples may be due to the types of prey consumed by the felines. As demonstrated, *L. guttulus* can feed on both *A. montensis* and *O. nigripes*, and the sizes of these wild rodents fall within the preferred prey sizes for the Atlantic Forest tiger-cat.

Thus, considering the presented phylogenetic analysis and the feeding preference of *L. guttulus* for *A. montensis*, *O. nigripes*, and other small rodents, it is possible that the Anelloviridae fragments were detected in the rectal swab due to the feeding habits of the Atlantic Forest tiger-cat.

## 5. Conclusions

This study of viral diversity in wild animals from the Sinos River Valley revealed a diverse range of viral families across species. Notably, it marks the first report of a Chikungunya virus-like (ChikV) in the rodent species *Akodon montensis* in Brazil, as well as in *Coendou spinosus*. The detection of ChikV, along with other viral families, underscores the importance of genomic surveillance in a One Health framework, emphasizing the interconnectedness of human, animal, and environmental health in pathogen emergence and transmission. Also, these findings provide valuable insights into the ecological and epidemiological dynamics of these viruses.

Understanding viral circulation in wildlife is critical for anticipating spillover events and mitigating zoonotic risks. Ongoing research into potential reservoirs and vectors, even in species not traditionally considered hosts, is essential for characterizing transmission dynamics and evaluating public health threats. Early detection and the continuous monitoring of emerging viruses in wildlife populations are key to preventing outbreaks and limiting disease spread. Integrating multidisciplinary approaches that consider ecological changes, habitat disturbances, and human-wildlife interactions is fundamental to strengthening disease surveillance and response strategies within a One Health paradigm.

## Figures and Tables

**Table 1:** Municipalities and locations where the animals were collected, as well as the number of individuals collected for each species.

Total Number of Samples		Animals Collected	Common Name	Species	Geographic Coordinates	Location
Oral Swabs	Rectal Swabs					
-	01/01	01	*Nutria*	*Myocastor coypus*	−29.761097 −51.042227	Novo Hamburgo
-	01/01	01	*Atlantic Forest tiger-cat*	*Leopardus guttulus*	−29.6273186 −51.1128742	Dois Irmãos
01/01	01/01	01	*White-tufted-ear Marmoset*	*Callithrix jacchus*	−29.688729 −51.108325	Novo Hamburgo
01/01	01/01	01	*Ihering’s Three-striped Opossum*	*Monodelphis iheringi*	−29.652316 −50.576316	Rolante
01/01	01/01	01	*Small wild rodent*	*Brucepattersonius iheringi*	−29.652316 −50.576316	Rolante
01/01	01/01	01	*Small wild rodent*	*Thaptomys nigrita*	−29.652316 −50.576316	Rolante
01/01	01/01	01	*Small wild rodent*	*Sooretamys angouya*	−29.652316 −50.576316	Rolante
01/01	01/01	01	*Bat*	*Eptesicus furinalis*	−29.769097 −50.964491	Novo Hamburgo
01/01	01/01	01	*Bat*	*Lasiurus blossevillii*	−29.769097 −50.964491	Novo Hamburgo
04/17	17/17	17	*Bat*	*Molossus molossus*	−29.7215628 −51.0180754	Novo Hamburgo
15/15	15/15	15	*Small wild rodent*	*Akodon montensis*	−29.7407319 −51.0523446	Novo Hamburgo
01/04	04/04	04	*Porcupine*	*Coendou spinosus*	(a) −29.688729 −51.108325 (b) −29.681395 −51.109275	(a) Novo Hamburgo (b) Campo Bom
06/43	43/43	43	*Opossum*	*Didelphis* *Albiventris*	−29.688729 −51.108325	Novo Hamburgo

**Table 2:** Viral classes and families by species and kind of swab. The phages are separated below the table.

Species	Viral Class/Family	Swab
*Didelphis albiventris*	Autographiviridae	Rectal/Oral
	Togaviridae	Rectal
	Coronaviridae	Rectal/Oral
	Retroviridae	
	Flaviviridae	Rectal
	Orthoherpesviridae	Rectal/Oral
	Pneumoviridae	
	Adenoviridae	Rectal
	Smacoviridae	Rectal
*Coendou spinosus*	Togaviridae	Rectal/Oral
	Retroviridae	
*Leopardus guttulus*	Anelloviridae	Rectal
	Dicistoviridae	
	Parvoviridae	
*Eptesicus furinalis*	Retroviridae	Oral
*Sooretamys angouya*	Retroviridae	Oral
*Brucepattersonius iheringi*	Retroviridae	Oral
*Monodelphis iheringi*	Roboviria	Rectal
	Orthomyxoviridae	Oral
*Akodon montensis*	Togaviridae	Rectal/Oral
	Retroviridae	
	Marseilleviridae	
	Autographiviridae	Oral
	Adenoviridae	Oral
*Molossus molossus*	Orthoherpesviridae	Rectal
	Retroviridae	
	Coronaviridae	
*Lasiurus blossevillii*	Coronaviridae	Oral
**Species**	**Phage Family or Class**	**Swab**
*Didelphis albiventris*	Microviridae	Rectal
	Caudovirecetes	Rectal/Oral
	Steigviridae	Rectal
	Schitoviridae	Rectal/Oral
	Herelleviridae	
	Peduoviridae	
	Chimalliviridae	
	Straboviridae	
	Suoliviridae	Rectal
*Coendou spinosus*	Caudovirecetes	Rectal/Oral
*Leopardus guttulus*	Microviridae	Rectal
	Casjensviridae	
*Sooretamys angouya*	Caudovirecetes	Oral
*Thaptomys nigrita*	Caudovirecetes	Rectal
	Peduoviridae	
*Callithrix jacchus*	Caudovirecetes	Rectal/Oral
*Monodelphis iheringi*	Caudovirecetes	Rectal/Oral
*Akodon montensis*	Straboviridae	Oral
	Peduoviridae	Rectal/Oral
	Caudovirecetes	
*Molossus molossus*	Caudovirecetes	Rectal/Oral
	Microviridae	

**Table 3:** Metagenomics data of the ChikV samples.

Depth Coverge	Coverage (%)	Reads	Contigs	Swab	Specie
9.7	98.92	1214	2	Rectal	*Coendou spinosus*
8.6	98.48	1097	1	Rectal	*Coendou spinosus*
74.8	99.3	10,084	1	Rectal	*Akodon montensis*
11.5	98.7	1473	1	Oral	*Akodon montensis*
15.5	98.64	2054	1	Rectal	*Akodon montensis*

## Data Availability

The FASTA files for Chikungunya Virus were submitted to the GISAID platform, and the Anellovirus was submitted to the GenBank platform. The accession numbers on the GISAID platform are EPI_ISL_19725188, EPI_ISL_19725187, EPI_ISL_19725186, EPI_ISL_19725185 and EPI_ISL_19725184. The accession number on the GenBank platform is PV112431.1. FASTQ files can be provided to interested parties upon request.

## Supplementary Materials

The following supporting information can be downloaded at: https://www.mdpi.com/article/10.3390/pathogens14040310/s1, Table S1: All the genera found by animal; Figure S1: The heatmap by viral genus created by CZ ID.

## Abbreviations

The following abbreviations are used in this manuscript:

CAPES	Coordination for the Improvement of Higher Education Personnel
CNPQ	Brazilian National Research Council
